# TNFα mediated ceramide generation triggers cisplatin induced apoptosis in B16F10 melanoma in a PKCδ independent manner

**DOI:** 10.18632/oncotarget.26478

**Published:** 2018-12-28

**Authors:** Sweta Ghosh, Junaid Jibran Jawed, Kuntal Halder, Sayantan Banerjee, Bidisha Paul Chowdhury, Akata Saha, Subir Kumar Juin, Suchandra Bhattacharyya Majumdar, Anamika Bose, Rathindranath Baral, Subrata Majumdar

**Affiliations:** ^1^ Division of Molecular Medicine, Bose Institute, Kolkata, West Bengal 700054, India; ^2^ Department of Immunoregulation and Immunodiagnostics, Chittaranjan National Cancer Institute (CNCI), Kolkata, West Bengal 700026, India

**Keywords:** melanoma, ceramide, PKCδ, tumor necrosis factor α, cisplatin

## Abstract

Ceramide is one of the important cellular components involved in cancer regulation and exerts its pleiotropic role in the protective immune response without exhibiting any adverse effects during malignant neoplasm. Although, the PKCδ-ceramide axis in cancer cells has been an effective target in reduction of cancer, involvement of PKCδ in inducing nephrotoxicity have become a major questionnaire. In the present study, we have elucidated the mechanism by which cisplatin exploits the ceramide to render cancer cell apoptosis leading to the abrogation of malignancy in a PKCδ independent pathway with lesser toxicity. Our study revealed that cisplatin treatment in PKCδ silenced melanoma cells induces ceramide mediated apoptosis. Moreover, cisplatin induced upregulation of the transcription factor IRF1 leading to the induction of the transcriptional activity of the TNFα promoter was evident from the pharmacological inhibition and RNA interference studies. Increased cellular expression of TNFα resulted in an elevated ceramide generation by stimulating acid-sphingomyelinase and cPLA_2_. Furthermore, reciprocity in the regulation of sphingosine kinase 1 (Sphk1) and sphingosine kinase 2 (Sphk2) during PKCδ independent ceramide generation was also observed during cisplatin treatment. PKCδ inhibited murine melanoma model showed reduction in nephrotoxicity along with tumor regression by ceramide generation. Altogether, the current study emphasized the unexplored signaling cascade of ceramide generation by cisplatin during PKCδ silenced condition, which is associated with increased TNFα generation. Our findings enlightened the detailed mechanistic insight of ceramide mediated signaling by chemotherapeutic drugs in cancer therapy exploring a new range of targets for cancer treatment strategies.

## INTRODUCTION

Malignant neoplasm, commonly known as cancer, is a well known life threatening disease and among its diverse classification melanoma is largely considered to be a chemotherapy refractory tumor [1, 2]. The treatment for metastatic melanoma is challenging while in the ample amount of cases, unsuccessful despite advances in ongoing medical research. Therefore, there is a need of detailed mechanistic study of cellular pathways related to available treatments [3–5]. In the past two decades, studies on sphingolipids revealed the important role of its bioactive forms such as ceramide, in the regulation of multiple biological functions especially in apoptosis [6, 7]. Inhibiting cell death by interference on ceramide signaling is a key strategy for tumorigenesis and escape from apoptosis stimuli [8, 9]. Therefore, for effective cancer therapy development, ceramide metabolic pathways have become a major concern. Anti-carcinogenic effect of ceramide in the treatment of malignancy depends on the stimulation of diverse apoptotic pathways.

Cisplatin, an effective platinum-containing anti-neoplastic agent is widely utilized in the treatment of a variety of cancers [10, 11], but its adverse effect due to its ability to generate toxicity in various tissue types is a major concern [12, 13]. While regulating the cellular signaling axis to trigger cell death, cisplatin also targets ceramide pathway to establish its utility in cancer deterioration through apoptosis [14]. There are other reports that ceramide production is regulated through protein kinase C (PKC) signaling pathway. PKCs are serine-threonine kinases, different isotypes of which play a key regulatory role in the proliferation, survival, or apoptosis of various cell types. Amongst different PKC isotypes, PKCδ induces apoptosis in cancer cell and plays a potential tumor suppressor role [15, 16]. It has also been reported that cisplatin mediated ceramide generation in cancer cells leads to tumor regression. Moreover, PKCδ plays a major role in ceramide generation in cancer cells leading to the induction of apoptosis [17, 18]. Furthermore, reports also suggest PKCδ as critical mediator of cisplatin induced kidney cell injury as well as death while inhibition of PKCδ retains chemotherapeutic efficacy of the drug with simultaneous abrogation of the nephrotoxicity [19, 20].

Previous studies from our laboratory reported that specific PKC isotypes were reciprocally regulated and utilized for enhancing the ceramide generation to induce apoptosis in the B16F10 melanoma cells [18]. In continuation with the exploration of the mechanism of endogenous ceramide generation, we investigated the role of cisplatin on the growth of mouse B16F10, human A375 melanoma cells *in vitro* and murine melanoma tumor *in vivo* under PKCδ deficient condition. Therefore, for the first time our study highlighted the cisplatin mediated inhibition of cancer cell growth in a PKCδ independent manner. Major focus of our study related to the apoptosis of melanoma cells is to understand the mechanism of ceramide generation by cisplatin in PKCδ deficient cell, while IRF-1 and TNFα emerged as key regulatory molecule.

Interferon regulatory factors (IRF) are transcription factors comprising of a large number of isoforms, among which IRF-1 and IRF-8 (or ICSBP) are associated with a vast range of host responses to infection and tumor growth [21–23]. On the other hand, TNFα is a pleiotropic cytokine that regulates a broad range of biological activities including cell differentiation, proliferation and death as well as inflammation and tissue development [24, 25]. Moreover, previous reports demonstrated that the expression of IRF-1, also known as interferon stimulated-gene factor 2 (ISGF-2), is synergistically induced by TNFα and IFNγ [26]. However, key enzymes involved in ceramide signaling pathway also include SphK1 and SphK2, which have distinct roles in sensitivity to cisplatin and other drugs modulation [27–29]. Relating these regulations, our study is majorly focused on the role of cisplatin induced apoptosis through PKCδ independent pathway involving different transcription factors and enzymes. Silencing of PKCδ retains the effect of cisplatin in hypoxic conditions, suggesting a novel regulation in hypoxia, which is an important selective force in the clonal evolution of tumors [30].

With such objectives in mind, the present work has highlighted the important cellular signaling events that sensitize PKCδ deficient melanoma cells towards proliferation inhibition and apoptosis by a *de novo* pathway. This pathway is also associated with increased generation of pro-inflammatory cytokine TNFα which may provide a useful therapeutic strategy to enhance the ability of cisplatin to eradicate tumors with lesser adverse effects.

## RESULTS

### Cisplatin inhibits cell cycle progression and induces apoptosis in PKCδ silenced B16F10 cells via ceramide generation

**Figure 1:**
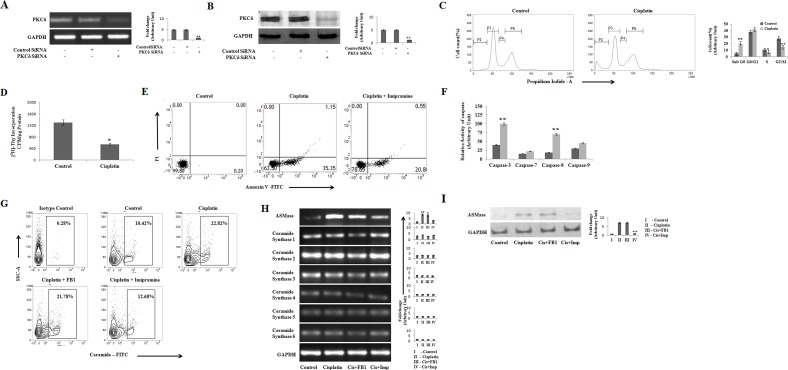
Cisplatin inhibits cell cycle progression and induces apoptosis in PKCδ silenced B16F10 cells via ceramide generation **(A)** 2 × 10^6^ B16F10 cells were transfected with PKCδ specific siRNA or control siRNA as mentioned in Materials and Methods. The transfected cells were collected in TRIZOL for mRNA expression of PKCδ by semi quantitative RT-PCR. GAPDH was used as a reference. Data are from one of the three representative experiments. Bar diagram is represented as mean ± SD. ^*^P < 0.05, ^**^P < 0.001 vs untreated. **(B)** In a separate experiment, similarly B16F10 cells were transfected with PKCδ specific siRNA or control siRNA as mentioned in Materials and Methods. The expression of PKCδ was analyzed in whole cell lysates by western blot analysis. GAPDH was used as a reference. Data are from one of three representative experiments. Bar diagram is represented as mean ± SD. ^*^P < 0.05, ^**^P < 0.001 vs untreated. **(C)** B16F10 cells transfected PKCδ specific siRNA with as mentioned in materials and methods were treated with cisplatin (50μM), harvested for 24 hrs and stained with propidium iodide to measure the DNA content by flow cytometry. **(D)** Cisplatin treated B16F10 cells transfected PKCδ specific siRNA were measured for incorporation of (3H) - thymidine to determine cell proliferation. Bar diagram is represented as mean ± SD. ^*^P < 0.05, ^**^P < 0.001 vs untreated. **(E)** PKCδ silenced B16F10 melanoma cells were treated with cisplatin (50μM) and cisplatin along with imipramine (10μM) for 12 hrs. The treated cells were collected and stained with Annexin-V FITC and PI and subsequently analyzed by flow cytometry. Data are from one of the three representative experiments. **(F)** B16F10 cells were transfected with PKCδ siRNA followed by cisplatin treatment. Caspase-3, 7, 8, 9 activities were determined from cell lysates using the caspase assay kits according to the manufacturer's instructions. Bar diagram is represented as mean ± SD. ^*^P < 0.05, ^**^P < 0.001 vs untreated. **(G)** PKCδ silenced B16F10 melanoma cells were treated with Cisplatin (50μM), cisplatin and FB1 (10μM), cisplatin and imipramine (10μM) for 12 hrs. The treated cells were subsequently stained with anti-mouse ceramide-FITC, isotype-matched control mouse antibody. Ceramide expression was analysed by flow cytometry. Data are from one of three representative experiments. **(H)** and **(I)** Similarly PKCδ specific siRNA transfected B16F10 melanoma cells were treated with cisplatin (50μM), cisplatin and FB1 (10μM), cisplatin and imipramine (10μM) for 12 hrs. The treated cells were collected in TRIZOL for mRNA expression analyses of ceramide synthase (1 – 6) and ASMase by semi quantitative RT-PCR. In a separate experiment, the expression of ASMase was analyzed in whole cell lysates by western blot analysis. (I) GAPDH was used as a reference. Data are from one of three representative experiments. Bar diagram is represented as mean ± SD. ^*^P < 0.05, ^**^P < 0.001 vs untreated.

Cisplatin, a well established chemotherapeutic agent, is involved in apoptosis of cancer cells and abrogate malignancy [10]. Cisplatin is also associated with high nephrotoxicity. Therefore, the mechanism of its action is the major area of concern [19]. It is established that ceramide is one of the major key players of cisplatin induced apoptosis, where PKCδ is a well-known modulator of cisplatin induced ceramide generation [14, 18]. However, recent studies have also depicted the involvement of TNFα in cisplatin induced apoptosis process [25]. Therefore, we were interested to investigate whether cisplatin could induce apoptosis of their target cells in a PKCδ independent manner. Accordingly, we silenced PKCδ in B16F10 cells using specific siRNA (Figure 1A and 1B) and the effect of cisplatin on cell cycle progression was studied. Interestingly, cisplatin at 50μM concentration showed a significant increase in the number of cells in sub G0/G1 phase and a concomitant decrease in the number of cells in S and G2/M phase, indicating that cisplatin halted G1-S transition resulting in cell cycle arrest and also gave rise to the sub G_0_/G1 cells from control to drug treatment, according to the morphological analysis these were apoptotic cells (Figure 1C). Cell proliferation analysis using (^3^H) – Thymidine incorporation assay revealed a significant decrease in the proliferation of cisplatin treated cells (Figure 1D). In order to check the translocation of phosphatidylserine (PS) externalization from inner cell membrane to outer cell membrane, a characteristic feature of cells undergoing apoptosis, cells were subjected to flow cytometric analysis after staining with Annexin-V-FITC and PI. The percentage of apoptosis in cisplatin treated cells was found to be 37.95% as compared to a very low percentage of apoptotic cells in the untreated PKCδ deficient B16F10 melanoma cells (Figure 1E). On analyzing different caspase activities upon cisplatin mediated apoptosis, caspase-3 and caspase-8 activities were found to be elevated significantly, which confirms their role in cisplatin mediated apoptosis (Figure 1F). Moreover, we wanted to know whether PKCδ deficient melanoma cells could generate ceramide upon cisplatin treatment. We observed that cisplatin significantly increased the level of ceramide (Figure 1G). In order to determine the pathway of ceramide generation, PKCδ deficient B16F10 melanoma cells were treated with FB1 (10μM) or imipramine (10μM), the well-known inhibitors of *de novo* and ASMase pathway respectively following cisplatin treatment. It was observed that in the presence of imipramine, cisplatin failed to induce ceramide generation (Figure 1G). Furthermore, the mRNA expression of ASMase was upregulated by cisplatin treatment, which was also reduced by imipramine treatment (Figure 1H). Interestingly the six isoforms of ceramide synthase, the key enzymes of the *de novo* synthesis pathway, showed no effect on the cisplatin mediated ceramide generation (Figure 1H). Taken together, our results confirmed that the ceramide which is produced by sphingomyelinase pathway is involved in cisplatin mediated apoptosis of PKCδ deficient B16F10 melanoma cells.

### Cisplatin induces apoptosis in PKCδ silenced B16F10 cells by TNFα mediated pathway

**Figure 2:**
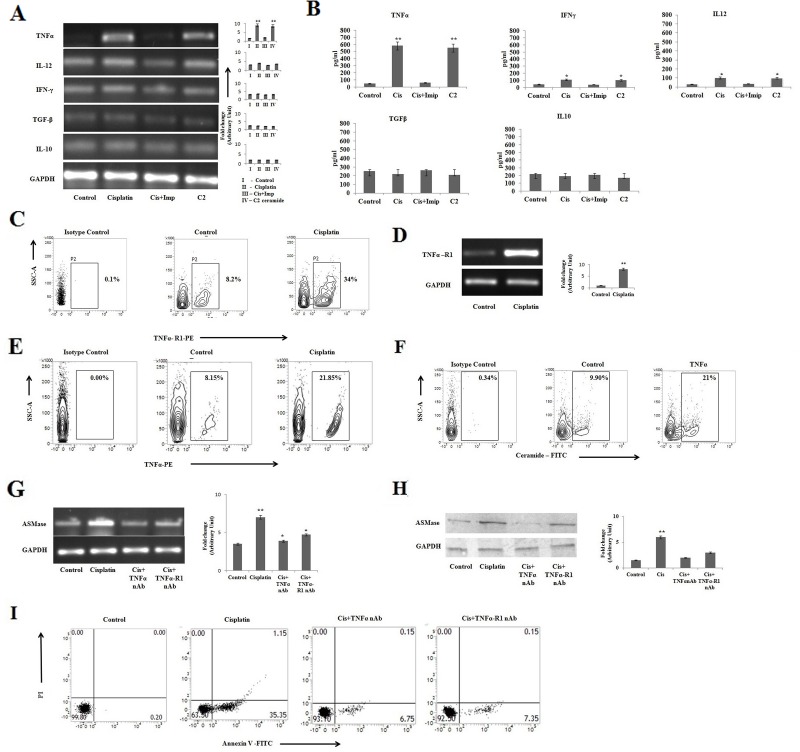
Cisplatin induces apoptosis in PKCδ silenced B16F10 cells by TNFα mediated pathway **(A)** and **(B)** B16F10 cells were transfected with PKCδ siRNA followed by cisplatin, cisplatin along with impiramine, and C2 ceramide treatment. The treated cells were collected in TRIZOL for mRNA expression analyses of TNF-α, IL-12, IFN-γ, IL-10 and TGF-β by semi quantitative RT-PCR. The cell supernatants were collected for estimation of TNF-α, IL-12, IFN-γ, IL-10 and TGF-β by ELISA. GAPDH was used as a reference. Data are from one of three representative experiments. Bar diagram is represented as mean ± SD. ^*^P < 0.05, ^**^P < 0.001 vs untreated. **(C)** PKCδ silenced B16F10 melanoma cells were treated with Cisplatin followed by fixation and staining for FITC-conjugated TNF-R1 expression and analyzed by flow cytometry. Data are from one of three representative experiments. **(D)** In a separate experiment, similarly B16F10 cells transfected with PKCδ specific siRNA were treated with cisplatin and collected in TRIZOL for mRNA expression analyses of TNF-R1 by semiquantitative RT-PCR. GAPDH was used as a reference. Data are from one of the three representative experiments. Bar diagram is represented as mean ± SD. ^*^P < 0.05, ^**^P < 0.001 vs untreated. **(E)** PKCδ silenced B16F10 melanoma cells were treated with cisplatin and then the cells were subsequently stained for intracellular cytokine staining with anti-mouse TNFα-PE, isotype-matched control mouse antibody. TNFα expression was analysed by flow cytometry. Data are from one of the three representative experiments. **(F)** PKCδ silenced B16F10 melanoma cells were treated with exogenous TNFα and then the cells were subsequently stained with anti-mouse ceramide-FITC, isotype-matched control mouse antibody. Ceramide expression was analysed by flow cytometry. Data are from one of three representative experiments. **(G)** and **(H)** Similarly PKCδ specific siRNA transfected B16F10 melanoma cells were treated with cisplatin, cisplatin along with TNFα or TNFα-R1 neutralizing antibody. The treated cells were collected in TRIZOL for mRNA expression of ASMase by semi quantitative RT-PCR. GAPDH was used as a reference. Data are from one of the three representative experiments. Bar diagram is represented as mean ± SD. ^*^P < 0.05, ^**^P < 0.001 vs untreated. **(I)** In a separate experiment PKCδ specific siRNA transfected B16F10 melanoma cells were treated with cisplatin, cisplatin and TNFα neutralizing antibody together and cisplatin along with TNFα-R1 neutralizing antibody. The treated cells were collected and stained with Annexin-V FITC and PI and subsequently analyzed by flow cytometry. Data are from one of the three representative experiments.

Cytokines released in response to inflammation and immunity can function to inhibit cancer development and progression [31]. Cytokines with various biological effects act as autocrine or paracrine factors and they are capable in modulation of tumor growth [32]. On the other hand, malignant cells can act in response to a few cytokines, which in turn support growth, knock down apoptosis, and assist invasion via metastasis. A more detailed understanding of cytokine-cancer cell interactions provides new opportunities for improving cancer immunotherapy [33]. Therefore, we investigated the status of different cytokines in cisplatin mediated apoptosis of PKCδ deficient B16F10 cells. It was observed that cisplatin in PKCδ deficient B16F10 melanoma cells were unable to induce the expression of IL-12 and IFN-γ (Figure 2A), whereas it increased the expression of TNFα. The expression of anti-inflammatory cytokines, including TGF-β and IL-10 remained unaltered. In all the cases, C2 ceramide (20μM) at 12 h was taken as positive control and the regulatory effect of ceramide on various cytokines was further confirmed by ELISA (Figure 2B).

Cisplatin treatment also increased the expression of TNFα receptor which was confirmed by FACS and PCR analyses (Figure 2C and 2D). Moreover, cisplatin treatment increased the production of intracellular TNFα in PKCδ deficient B16F10 cells compared to the untreated cells (Figure 2E). The effect of exogenous recombinant TNFα (50ng/ml) on ceramide generation (Figure 2F) and the effect of TNFα as well as TNFα-R1 neutralizing antibody (10 μg/ml) on ASMase expression in relation to mRNA (Figure 2G), protein level (Figure 2H) and apoptosis (Figure 2I) further confirmed that cisplatin mediated ceramide generation is TNFα and ASMase dependent.

### Cisplatin induces IRF-1 dependent TNFα activation for the initiation of apoptosis in PKCδ silenced B16F10 melanoma cells

**Figure 3:**
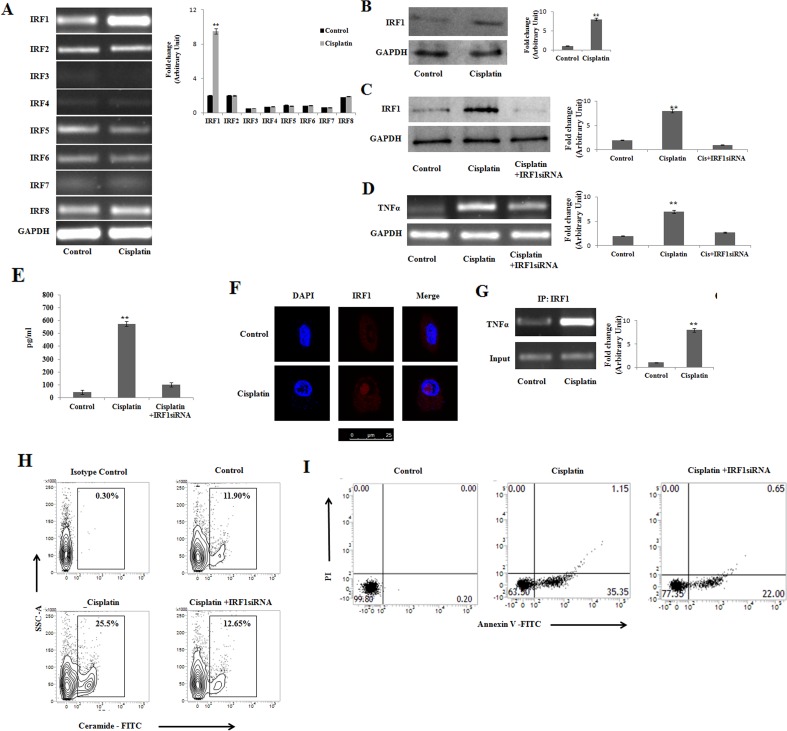
Cisplatin induces IRF-1 dependent TNFα activation for initiation of apoptosis in PKCδ silenced B16F10 melanoma cells **(A)** and **(B)** B16F10 cells were transfected with PKCδ siRNA followed by Cisplatin treatment. The treated cells were collected in TRIZOL for mRNA extraction and semi quantitative RT-PCR analyses for IRF 1- 8 were done. The expression IRF 1 was analyzed in whole cell lysates by Western blotting. GAPDH was used as a reference. Data are from one of three representative experiments. Bar diagram is represented as mean ± SD. ^*^P < 0.05, ^**^P < 0.001 vs untreated. **(C)** PKCδ silenced and cisplatin treated B16F10 cells were transfected with IRF1 specific siRNA mentioned in materials and methods and harvested for 24 hrs. The expression with IRF 1 was analysed in whole cell lysates by western blot analysis. GAPDH was used as a reference. Data are from one of the three representative experiments. Bar diagram is represented as mean ± SD. ^*^P < 0.05, ^**^P < 0.001 vs untreated. **(D)** and **(E)** In a separate experiment, similarly PKCδ silenced and cisplatin treated B16F10 cells were transfected with IRF1 specific siRNA as mentioned in Materials and Methods. The treated cells were collected in TRIZOL for TNFα mRNA expression by semiquantitative RT-PCR. GAPDH was used as a reference. The cell supernatants were collected for the estimation of TNFα by ELISA. Data are from one of the three representative experiments. Bar diagram is represented as mean ± SD. ^*^P < 0.05, ^**^P < 0.001 vs untreated. **(F)** PKCδ silenced and cisplatin treated B16F10 cells were stained with PE- IRF-1 and analyzed by confocal microscopy. IRF-1 sub cellular localization was studied by confocal microscopy using the anti mouse IRF-1 monoclonal antibody and DAPI for nuclear staining. Scale bar 25 μm. Data are from one of the three representative experiments. **(G)** B16F10 cells were transfected with PKCδ siRNA followed by Cisplatin treatment. In treated cells IRF1 binding at TNFα promoter (IP: IRF1), was analyzed by ChIP assay as described in materials and methods. Data are from one of the three representative experiments. Bar diagram is represented as mean ± SD. ^*^P < 0.05, ^**^P < 0.001 vs untreated. **(H)** PKCδ silenced and cisplatin treated B16F10 cells were transfected with IRF1 specific siRNA mentioned in materials and methods and harvested for 24 hrs. Then the cells were subsequently stained with anti-mouse ceramide-FITC, isotype-matched control mouse antibody. Ceramide expression was analysed by flow cytometry. Data are from one of the three representative experiments. **(I)** In a separate experiment, similarly PKCδ silenced and cisplatin treated B16F10 cells were transfected with IRF1 specific siRNA as mentioned in Materials and Methods. Cells were collected and stained with Annexin-V FITC and PI and subsequently analyzed by flow cytometry. Data are from one of the three representative experiments.

The cytokine responsiveness depends on interferon regulatory factors (IRFs) expression [26], which in turn can be induced by cisplatin [34]. IRFs can also control tumor progression by regulating the interactions between cancer and immune cells within the tumor microenvironment [35]. Therefore, we examined the effect of cisplatin on the expression of these IRFs in PKCδ deficient B16F10 cells. It was observed that cisplatin differentially modulated the expression of eight IRFs in PKCδ deficient B16F10 (Figure 3A). It was interesting to observe that among the eight IRFs, only the expression of IRF1 was significantly increased compared to the other IRFs upon cisplatin treatment. To further confirm the involvement of cisplatin in the regulation of IRF1, we performed western blot analysis which revealed the similar pattern of expression corroborating the previous findings (Figure 3B). Our results indicated that silencing IRF1 expression abrogated cisplatin mediated increase in IRF1 (Figure 3C) along with TNFα expression (Figure 3D) and protein level (Figure 3E) and ceramide generation (Figure 3H).

In order to determine the role of IRF1 in cisplatin induced variations in expression, an *in silico* analysis was performed to evaluate the possible enrichment of binding sites for transcription factors in the putative promoter regions of the gene subgroups that were differentially regulated by cisplatin. For that we used the free web tool oPOSSUM. The result showed that cisplatin can increase IRF1 expression (data not shown). To further confirm the involvement of IRF1 in response to cisplatin, the localization of IRF1 was studied by confocal microscopy (Figure 3F). Our result showed that upon cisplatin treatment IRF1 nuclear translocation was increased in comparison with the control set. Moreover, a prominent binding of IRF1 to the TNFα promoter was observed in PKCδ deficient B16F10 cells (Figure 3G). Further, FACS analysis of Annexin-V assay using IRF1 siRNA confirmed the role of IRF1 in cisplatin mediated apoptosis (Figure 3I). Thus, our finding suggests that IRF1 regulates TNFα mediated ceramide generation in PKCδ deficient B16F10 cells.

### cPLA_2_ modulates cisplatin mediated apoptosis in PKCδ silenced B16F10 melanoma cells

**Figure 4:**
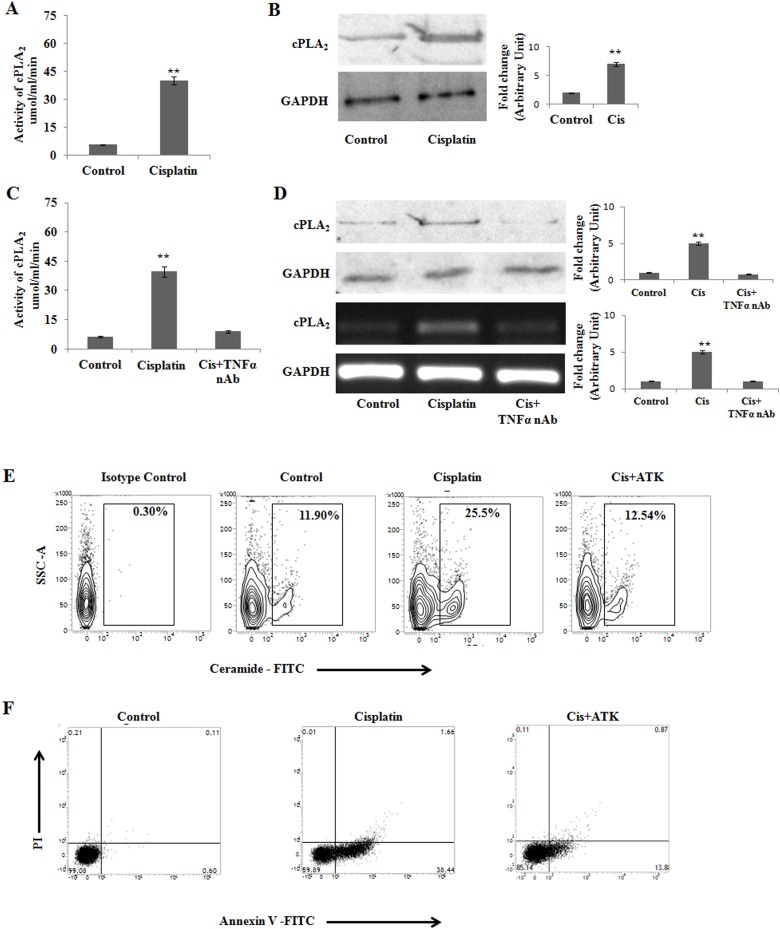
cPLA_2_ modulates cisplatin mediated apoptosis in PKCδ silenced B16F10 melanoma cells **(A)** and **(B)** B16F10 cells were transfected with PKCδ siRNA followed by Cisplatin treatment. cPLA_2_ activity was determined from cell lysates using the cytosolic phospholipase A2 assay kit according to the manufacturer's instructions. Bar diagram is represented as mean ± SD. ^*^P < 0.05, ^**^P < 0.001 vs untreated. The expression cPLA_2_ was analysed in whole cell lysates by western blot analysis. GAPDH was used as a reference. Data are from one of three representative experiments. Bar diagram is represented as mean ± SD. ^*^P < 0.05, ^**^P < 0.001 vs untreated. **(C)** PKCδ specific siRNA transfected B16F10 melanoma cells were treated with cisplatin and TNFα neutralizing antibody. cPLA_2_ activity was determined from cell lysates using the cytosolic phospholipase A2 assay kit according to the manufacturer's instructions. Bar diagram is represented as mean ± SD. ^*^P < 0.05, ^**^P < 0.001 vs untreated. **(D)** Similarly PKCδ specific siRNA transfected B16F10 melanoma cells were treated with cisplatin and TNFα neutralizing antibody. The expression cPLA_2_ was analysed in whole cell lysates by western blotting. In a separate experiment, the treated cells were collected in TRIZOL for mRNA expression of cPLA_2_ by semi quantitative RT-PCR. GAPDH was used as a reference. Data are from one of three representative experiments. Bar diagram is represented as mean ± SD. ^*^P < 0.05, ^**^P < 0.001 vs untreated. **(E)** and **(F)** PKCδ silenced and cisplatin treated B16F10 cells were incubated in the presence or absence of the cPLA_2_ inhibitor ATK. Cells were collected and subsequently stained with anti-mouse ceramide-FITC, isotype-matched control mouse antibody. Ceramide expression was analysed by flow cytometry. In a different experiment, as mentioned in Materials and Methods, cells were stained with Annexin-V FITC and PI and subsequently analyzed by flow cytometry. Data are from one of the three representative experiments.

TNFα amplifies ceramide generation by activating two different intracellular signaling cascades involving ASMase (Figure 2F) as well as cytosolic phospholipase A2 (cPLA2) in PKCδ deficient B16F10 melanoma cells. The activity of cPLA_2_ is dependent on some proinflamatory cytokines in tumors [36, 37]. As cPLA_2_ is necessary for TNFα induced ceramide generation [38, 39], we investigated whether cisplatin treatment can regulate the cPLA_2_ expression. On analyzing cPLA2 enzyme activity upon cisplatin treatment (Figure 4A), cPLA2 activity was found to be elevated significantly. Similar observation was obtained in western blot analysis (Figure 4B). However, upon treatment with TNFα neutralizing (10 μg/ml) antibody, the cPLA2 activity was found to be decreased as confirmed by activity assay (Figure 4C), western blot as well as mRNA expression analysis (Figure 4D).

Moreover, when 10μM arachidonyl trifluoromethyl ketone (ATK; a cPLA2 inhibitor) is treated in PKCδ deficient B16F10 melanoma, it failed to augment ceramide generation (Figure 4E) and apoptosis (Figure 4F) in cisplatin treated cells. Therefore, our results clearly suggest that cPLA2 and ASMase mediated ceramide generation plays a major role in PKCδ deficient melanoma cells to induce apoptosis.

### Reciprocal regulation of Sphingosine kinase 1 and Sphingosine kinase 2 on cisplatin treated PKCδ silenced B16F10 melanoma cells

**Figure 5:**
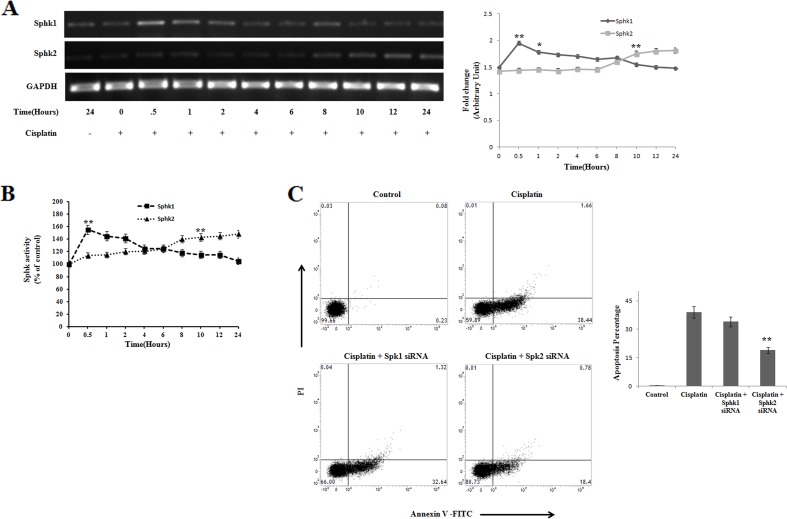
Reciprocal regulation of Sphingosine kinase 1 and Sphingosine kinase 2 on cisplatin treated PKCδ silenced B16F10 melanoma cells **(A)** B16F10 cells were transfected with PKCδ siRNA followed by cisplatin treatment in a time dependent manner. At different time points the treated cells were collected in TRIZOL for Sphk1 and Sphk2 mRNA expression by semi quantitative RT-PCR analysis. GAPDH was used as a reference. Data are from one of the three representative experiments. At different time points, data were represented as mean ± SD. ^*^P < 0.05, ^**^P < 0.001 vs untreated. **(B)** B16F10 cells were transfected with PKCδ siRNA followed by cisplatin treatment. Activities of sphingosine kinase 1 and sphingosine kinase 2 were determined from cell lysates following the protocol described in Materials and Methods. Bar diagram is represented as mean ± SD. ^*^P < 0.05, ^**^P < 0.001 vs untreated. **(C)** In a separate experiment, similarly PKCδ silenced cisplatin treated B16F10 cells were transfected with Sphk1 and Sphk2 specific siRNAs as mentioned in Materials and Methods. Cells were collected stained with Annexin-V FITC and PI and subsequently analyzed by flow cytometry. Data are from one of the three representative experiments.

Sphingosine kinase 1 and Sphingosine kinase 2 are two important mediators in ceramide induced apoptosis, where the fine tuning of both the enzymes decides the fate of cell [27, 28]. Activators of Sphk2 act as the promising regulator of apoptosis [40]. Sphk1 and Sphk2 maintained a reciprocal regulation in PKCδ silenced B16F10 melanoma cells in a time dependent manner (Figure 5A), which was found to be correlated with the activity assay of sphingosine kinases (Figure 5B). In the initial hrs of cisplatin treatment Sphk1 was found to be upregulated whereas the expression of Sphk2 was downregulated. In contrary, these expressions were reversed at later time points, indicating the reciprocal regulation of both the enzymes. To further confirm the role of Sphk1 and Sphk2 in the regulation of apoptosis, we performed FACS analysis using their respective siRNAs. The result showed that Sphk2 is majorly responsible for apoptosis in comparison with Sphk1 (Figure 5C). Therefore, our findings indicate that Sphk1 inhibition and Sphk2 activation are important for cisplatin mediated apoptosis in PKCδ deficient B16F10 melanoma cell.

### Cisplatin continues to induce apoptosis by ceramide generation during hypoxic condition in PKCδ silenced B16F10 cells

**Figure 6:**
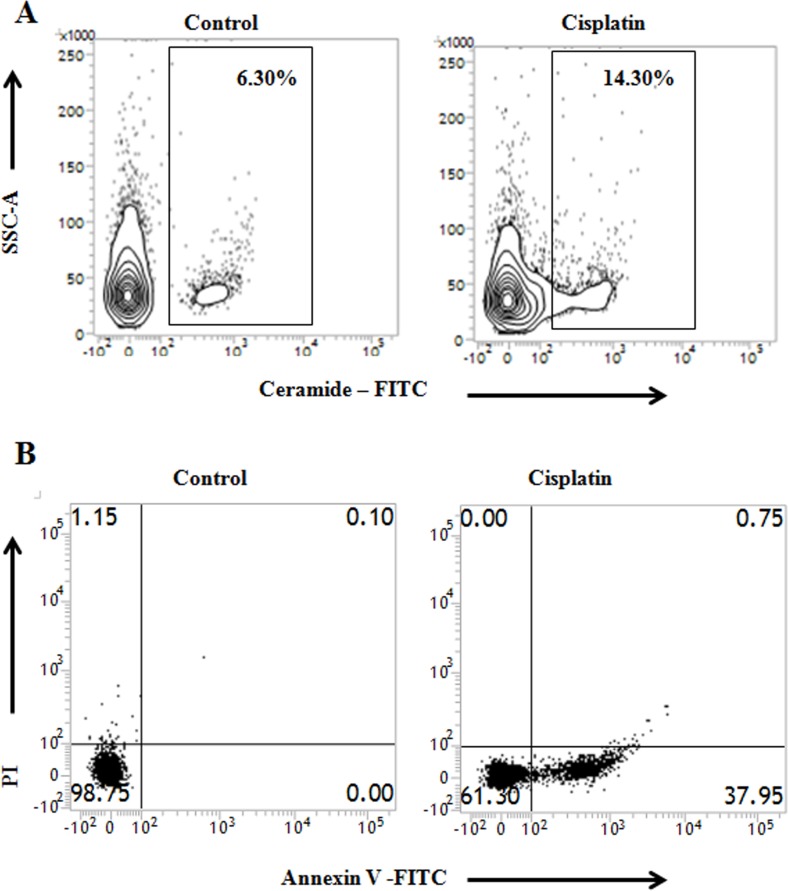
Cisplatin continues to induce apoptosis by ceramide generation during hypoxic condition in PKCδ silenced B16F10 Cells **(A)** and **(B)** B16F10 cells transfected with PKCδ siRNA followed by cisplatin treatment were cultured in hypoxic chamber as described in Materials and Methods. Cells were collected and subsequently stained with anti-mouse ceramide-FITC, isotype-matched control mouse antibody. Ceramide expression was measured by flow cytometry analysis. In a different experiment as mentioned in Materials and Methods, cells were stained with Annexin-V FITC and PI and subsequently analyzed by flow cytometry. Data are from one of the three representative experiments. Bar diagram is represented as mean ± SD. ^*^P < 0.05, ^**^P < 0.001 vs untreated.

Major tumor environmental regulations are prominent in tumor core, where the extent of hypoxia is more powerful than the periphery. So, generation of hypoxic conditions *in vitro* can successfully mimic the tumor core regulations [41, 42]. To evaluate the effect of cisplatin in PKCδ deficient conditions and to correlate it with tumor hypoxia, PKCδ deficient B16F10 melanoma cells were incubated in hypoxia chamber, where oxygen tension was maintained at 90% lower than normoxic environment. Exposure of cisplatin treated PKCδ deficient B16F10 melanoma cells to hypoxia continued to generate ceramide (Figure 6A) and induced apoptosis (Figure 6B). Hence, these results clearly demonstrated that cisplatin treatment has a great potential in melanoma therapy even in PKCδ deficient conditions.

### Cisplatin treatment in PKCδ silenced A375 human melanoma cells induces ceramide mediated apoptosis through IRF1-TNFα axis

**Figure 7:**
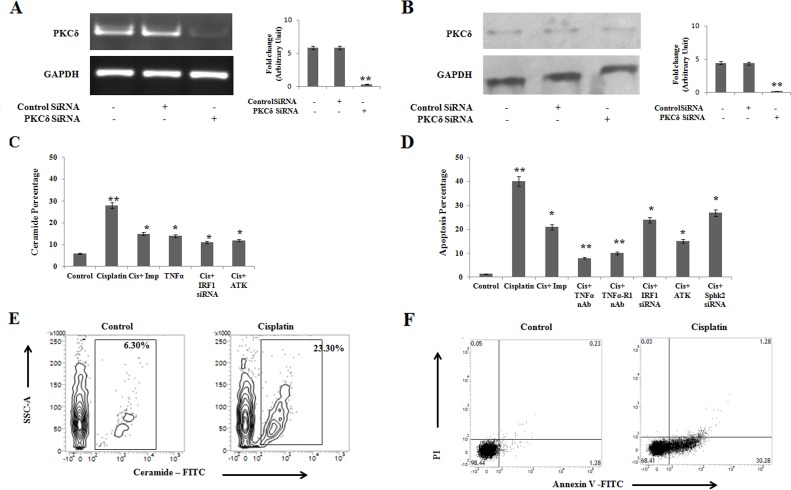
Cisplatin treatment in PKCδ silenced A375 human melanoma cells induces ceramide mediated apoptosis through IRF1-TNFα axis **(A)** 2 × 10^6^ A375 cells were transfected with PKCδ specific siRNA or control siRNA as mentioned in Materials and Methods and the transfected cells were collected in TRIZOL for mRNA expression of PKCδ by semi quantitative RT-PCR. GAPDH was used as a reference. Data are from one of the three representative experiments. Bar diagram is represented as mean ± SD. ^*^P < 0.05, ^**^P < 0.001 vs untreated. **(B)** In a separate experiment, similarly A375 cells were either transfected with PKCδ specific siRNA or control siRNA as mentioned in Materials and Methods. The expression of PKCδ was analyzed in whole cell lysates by western blot analysis. GAPDH was used as a reference. Data are from one of the three representative experiments. Bar diagram is represented as mean ± SD. ^*^P < 0.05, ^**^P < 0.001 vs untreated. **(C)** PKCδ silenced A375 cells were either treated with cisplatin alone or in combination with imipramine, ATK or transfected with IRF1 specific siRNAs. Cells were also treated with exogenous TNFα in absence of cisplatin. Treated cells were subsequently stained with anti-mouse ceramide-FITC, isotype-matched control mouse antibody. Ceramide expression was analysed by flow cytometry. Data are from one of the three representative experiments. Bar diagram is represented as mean ± SD. ^*^P < 0.05, ^**^P < 0.001 vs untreated. **(D)** PKCδ silenced A375 cells were either treated with cisplatin alone or in combination with imipramine, ATK, TNFα/TNFα-R1 neutralizing antibody or transfected with IRF1/Sphk2 specific siRNAs as mentioned in Materials and Methods. Cells were collected, stained with Annexin-V FITC and PI and subsequently analyzed by flow cytometry. Bar diagram is represented as mean ± SD. ^*^P < 0.05, ^**^P < 0.001 vs untreated. **(E)** and **(F)** A375 cells, transfected with PKCδ siRNA followed by cisplatin treatment were cultured in hypoxic chamber as described in Materials and Methods. Cells were collected and subsequently stained with anti-mouse ceramide-FITC, isotype-matched control mouse antibody. Ceramide expression was analysed by flow cytometry. In a different experiment cells were stained with Annexin-V FITC and PI and subsequently analyzed by flow cytometry. Data are from one of the three representative experiments.

Our previous results suggest a positive regulatory role of IRF1-TNFα interactions in ceramide generation leading to apoptosis in PKCδ silenced B16F10 mouse melanoma cells. Therefore, we intended to validate the role of TNFα signaling on ceramide generation pathway in PKCδ silenced A375 human melanoma cells. In the present study, after successfully silencing PKCδ in A375 cells using specific siRNA (Figure 7A and 7B) the effect of cisplatin on apoptosis was studied. We observed a marked decrease in the ceramide mediated apoptosis in cisplatin (50μM, 24h) treated PKCδ silenced A375 cells when imipramine (10μM), ATK (10μM) or IRF1 siRNA were used in the experiment (Figure 7C and Figure 7D). The treatment of exogenous recombinant TNFα (50ng/ml) also increased ceramide generation in PKCδ silenced A375 melanoma cells (Figure 7C). Interestingly, Sphk2 siRNA, TNFα or TNFα-R1 neutralizing antibody (10 μg/ml) treated cells showed higher percentage of apoptosis upon cisplatin treatment in comparison with the untreated cells (Figure 7D). On the other hand, under hypoxic condition, cisplatin was able to induce apoptosis (Figure 7F) by ceramide generation (Figure 7E) in A375 melanoma cells under PKCδ silenced condition. Hence our data suggests that the TNFα mediated signaling is also operational to increase apoptosis by ceramide generation in PKCδ silenced human melanoma cells.

### Effect of cisplatin treatment in PKCδ inhibited melanoma tumor growth *in vivo*

**Figure 8:**
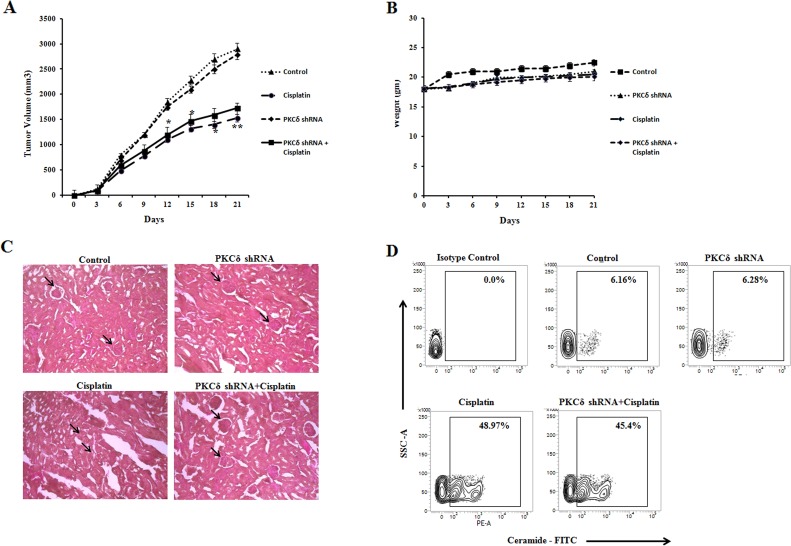
Effect of cisplatin treatment in PKCδ inhibited melanoma tumor growth in vivo **(A)** and **(B)** 3 × 10^5^ B16F10 melanoma cells were injected subcutaneously into the right flank of control and lentivirally PKCδ silenced female C57BL/6 mice and were left untreated or treated with cisplatin. The tumor volumes were monitored on every two days and the final tumor volume was determined at 21days post injection of tumor cells. The data shown here are representative of the three independent experiments. n = 4/ mice group. Bar diagram is represented as mean ± SD. ^*^P < 0.05, ^**^P < 0.001 vs untreated. **(C)** Representative kidney sections with or without PKCδ inhibited cisplatin treated mice groups were stained with hematoxylin and eosin (H&E). Arrows indicate conditions of glomerulus. Original magnification is 200X. **(D)** After enzymatic digestion of tumor, single cells were isolated and subsequently stained with anti-mouse ceramide-FITC, isotype-matched control mouse antibody. Ceramide expression was analysed by flow cytometry. Data are from one of the three representative experiments.

To evaluate the effect of cisplatin treatment *in vivo* in PKCδ inhibited B16F10 melanoma tumor growth, B16F10 murine models were developed. We investigated whether the lentiviral expression of PKCδ shRNA would reduce the toxicity of cisplatin treatment in C57BL/6 mice. Tumor-bearing normal mice, lentivirally silenced with control shRNA or PKCδ shRNA mediated mice were treated with PBS or cisplatin (10 mg/kg bw) for 7 days after day 3 of tumor cell inoculation. Animals were sacrificed when the tumors reached a palpable size at day 21. Similar responses were observed in the control shRNA treated mice as well as in mice with no shRNA treatment in all the experiments (data of control shRNA treated mice not shown). As shown in (Figure 8A), cisplatin significantly reduced PKCδ inhibited B16F10 tumor growth in comparison with untreated tumor. Moreover, the cisplatin treatment in PKCδ inhibited B16F10 tumor bearing mice did not reduce the body weight, suggesting that PKCδ inhibition has no significant toxicity (Figure 8B). Interestingly, we observed a reduction in the cisplatin mediated nephrotoxicity in kidney sections under PKCδ inhibited mice in comparison with uninhibited groups (Figure 8C). Control sets revealed normal glomeruli in the kidney section whereas cisplatin treatment revealed distorted glomerular kidney along with severe tubular damages. It is noteworthy that the cisplatin treatment in PKCδ silenced group was able in restoration of the normal glomerular kidney. Furthermore, the tumor samples of mice bearing B16F10 cells were isolated and experiments were conducted to determine whether cisplatin was able to generate ceramide *in vivo*. However, samples from mice treated with cisplatin for both PKCδ inhibited and uninhibited condition showed higher percentage of ceramide generation (Figure 8D). Hence, these results demonstrated that the cisplatin treatment under PKCδ inhibited condition could reduce ceramide mediated tumor growth with lesser toxic effects.

## DISCUSSION

The complete cure of metastatic melanoma is still a challenge whereas surgery and radiation therapy can only play a role in the palliation of symptoms [2–5]. One of the major reasons for the difficulty in metastatic melanoma treatment is its heterogeneity [43]. Henceforth detailed investigation of signal transduction and the importance of apoptosis as the crucial player of tissue homeostasis maintenance, have gained more attention. Our study deals with this challenge revealing new details about the mode of action of cisplatin.

From decades cisplatin has been consumed as a traditional chemotherapeutic medicine for its antitumor activity and still remains as a significant interest [10]. As chemotherapeutic approaches may have a palliative benefit, the effects of which in the modulation of natural cellular components during cancer regression are major topics of discussion. In relation with this, ceramide, the central molecule in sphingolipids metabolism can function as a tumor-suppressor lipid inducing antiproliferative and apoptotic responses in various cancer cells [6–9]. Conversely, S1P induced responses render S1P as a tumor-promoting lipid [44]. These discoveries are paving the way for the advancement of anticancer therapies. Ceramide mediated signaling is involved in diverse functioning. In *Leishmania* infection ceramide plays a negative role for host by creating a favorable environment for the parasite entry [45, 46], whereas in case of cancer, ceramide induces programmed cell death which in turn favors host survival. The modulations of ceramide signaling as an effective therapy have been reported against various types of cancer [8, 9]. Previous study from our laboratory reported that PKCδ over expression results in ceramide generation, which leads to cancer cell apoptosis [18]. Even exogenous ceramide administrations show anti-proliferative effects and has been associated with induction of apoptosis in many cancers [47]. Endogenous ceramide is produced in the cell via two distinct pathways i.e., *de novo* pathway and the salvage pathway depending on various stimuli. The salvage pathway of ceramide production needs sphingomyelinase activity for the generation of ceramide from sphingomyelin which is known as one of the key pathway for the regulation of apoptosis [7]. The salvage pathway of ceramide production is specifically regulated by some PKC isotype mediated sphingomyelinase activity. Report suggested that anticancer drug etoposide is capable of inducing PKCδ mediated ceramide generation by both *de novo* and neutral sphingomyelinase (nSMase) pathway in prostate cancer cell [48]. PKCs are serine/threonine kinases which play vivid roles in cell proliferation, apoptosis, differentiation and angiogenesis [16]. Previous reports suggested that cisplatin via PKCδ causes regression of tumor, but at the same time PKCδ is found to be the major reason behind cisplatin induced nephrotoxicity [19, 20]. In order to find out the effective therapy against melanoma, we investigated whether cisplatin is able to overcome protein kinase C dependency during generation of ceramide, which has a broad spectrum of antineoplastic actions with lesser adverse effects and a noticeable efficacy in a wide range of tumors. Initial experiments of our studies surprisingly revealed that cisplatin can induce ceramide generations in PKCδ independent manner via a novel pathway which involves increased generation of TNFα.

For the first time, we examined the effect of cisplatin treatment on proliferation of melanoma cells in PKC silenced conditions and interestingly, it was effective to reduce the cancer progression significantly. On the other hand, cisplatin also upregulated the ceramide generation via sphingomyalinase pathway by increasing ASMase expression, but the *de novo* pathway remained unaltered. Further studies revealed that increased ASMase expression is dependent on TNFα and TNFαR1 expression on cisplatin treatment in PKCδ silenced melanoma cells. When exogenous TNFα was administered, it continued to increase ceramide expression whereas its neutralization reduced the ASMase activity. Previous studies support our result that TNFα is mainly expressed by activated immune cells especially macrophages and T cells, but can also be produced by tumor cells and capable of exhibiting anticarcinogenic effect in various subtypes of cancer [49, 50].

Damages in DNA due to drugs, such as etoposide and doxorubicin result in the upregulation of the transcription factors like IRF-1, IRF-3 and IRF-7 [51–54]. IRF-1 was reported to play a significant role in modulation of the immune response, cell proliferation, differentiation and inflammation. Subsequently, it has been shown that IRF1 and IRF3 act as tumor suppressor genes by preventing oncogene mediated malignancy. While investigating the probable mediators of cisplatin induced TNFα elevation, IRF1 was diagnosed as a key transcription factor. IRF1 was found to be translocated to nucleus on cisplatin treatment and by binding to TNFα promoter it augmented TNFα production leading to enhanced ceramide generations. Apart from ASMase, cPLA_2_ was also found to be upregulated by TNFα and continued to magnify ceramide generation and corresponding apoptosis even in PKCδ silenced condition following cisplatin treatment. Earlier report suggested that PLA_2_ enzymes comprise a large and growing number of signal transduction enzymes [55] and these PLA_2_ isoforms can be induced by the inflammatory mediators like IL-1β and TNFα [56]. In addition, another two key enzymes of sphingolipid biosynthesis, Sphk1 and Sphk2 were found to get reciprocally regulated in a time dependent manner during cisplatin treatment. The role of two isoforms of sphingosine kinase (Sphk) is to catalyze the formation of sphingosine 1-phosphate, the key sphingolipid mediator [27]. Sphk1 stimulates cell growth and survival of cells whereas Sphk2 enhances apoptosis along with suppressed cellular proliferation [29, 40]. Our finding confirmed that Sphk2 causes apoptosis in PKCδ deficient B16F10 cells.

In advanced stage of cancer, hypoxic microenvironment plays a critical role in chemoresistance and it is the major mediator for various regulations in cancer [30, 57–59]. Hypoxia, which is majorly present in solid tumor, is generated due to the proliferation of tumor cells and outpaces the blood vessel formation in the tumor mass. To explore the effect of hypoxia during cisplatin induced apoptosis, we mimicked *in vivo* hypoxic tumor core conditions using hypoxic chamber. The results clearly indicated that cisplatin induced ceramide generation followed by apoptosis in PKCδ silenced conditions. Moreover, we have performed the key experiments in human A375 melanoma cells to increase the acceptability of our study. It was interesting to observe that the study on human melanoma cells revealed similar responses to the results obtained from the study carried out on B16F10 cells. Further, cisplatin administered PKCδ silenced murine melanoma model showed increased ceramide generation and simultaneous inhibition of tumor growth with reduced toxicity as revealed from the kidney sections. This finding validated our study in *in vivo* system. However, detailed mechanistic studies are required for better understanding of our findings in future.

**Figure 9:**
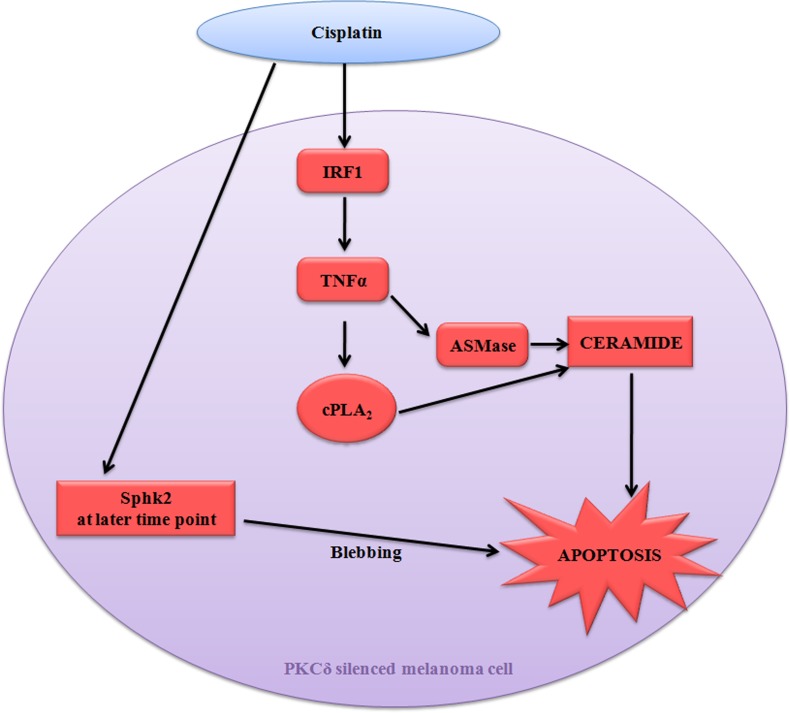
Diagrammatic representation of cisplatin mediated apoptosis of B16F10 melanoma cells in PKCδ independent manner

Taken together, our data decipher a novel mechanism exploited by cisplatin in PKCδ deficient conditions. It highlighted the involvement of bioactive sphingolipids ceramide and its impact to facilitate the host immune surveillance, restricting tumor growth through pro-inflammatory cytokine TNFα generation (Figure 9). These mechanistic details may open up a new insight of chemotherapy with lesser adverse effects exploiting natural cellular components like ceramide.

## MATERIALS AND METHODS

### Reagents and chemicals

DMEM medium, penicillin, streptomycin, L-glutamine, Cisplatin, AKT, Fumonisin B-1 (FB-1), Imipramine and Trizol were purchased from Sigma (St Louis, MO, USA). Fetal calf serum was from Gibco BRL (Grand Island, NY, USA). Deoxynucleoside triphosphates, RevertAid M-MuLV Reverse Transcriptase, oligo-dT, RNase inhibitor and other chemicals for cDNA synthesis were purchased from Fermentas (Ontario, Canada). Recombinant mouse TNFα was obtained from R&D (Minneapolis, Canada). Mouse TNFα, TNFα-R1 neutralizing antibody was from CST (Danvers, USA). Anti- cPLA_2_, PKCδ, Ceramide, IRF1, TNFα, TNFα-R1, GAPDH antibodies were purchased from Santa Cruz Biotechnology (San Jose, CA, USA). Mouse specific IRF1 small-interfering RNA (siRNA), human specific PKCδ, IRF1, Sphk2 siRNA and control siRNAs were obtained from Santa Cruz Biotechnology.

### Cell culture and transfection

B16F10 murine melanoma cells and A375 human melanoma cells were obtained from the National Centre for Cell Sciences, (Pune, India) and were cultured in Dulbecco's modified Eagle's medium (DMEM) supplemented with 10% fetal calf serum (FCS), 120 mg/ml penicillin, 200 mg/ml streptomycin and 2 mM L-glutamine. The cells were cultured in a humidified 5% CO_2_ incubator at 37°C. Cells were regularly cultured to maintain exponential growth of cells. Twenty-four hrs before transfection, cells were diluted in fresh medium without antibiotics and transferred to 24-well plates. Transient transfection of PKCδ siRNA was performed using a Lipofectamine PLUS reagent (Invitrogen, CA, USA) according to the manufacturer's recommendations. The efficiency of the transfection was monitored every 12 hrs. At 36 hrs, PKCδ expression was totally inhibited. Specific silencing was confirmed by at least three independent experiments. The cells were also treated with transfection reagent alone or with the non silencing scrambled PKCδ siRNA.

### Preparation of cell lysates

The adherent melanoma cell population was scraped and centrifuged at 2000 g for 10 mins at 4°C. The cells were then resuspended in ice-cold extraction buffer containing 50 mM Tris-HCl (pH-7.5), 50mM EGTA, protease inhibitor cocktail, and 50mM β-mercaptoethanol. Anti-protease mixture consisted of 0.33 mM leupeptin, 0.2 mM phenyl methyl sulfonyl fluoride (PMSF), and 0.35 mM of aprotinin. The cell containing suspension was sonicated at 4°C and centrifuged at 8000g for 15 mins at 4°C. The supernatant was used for experiments after determination of protein content.

### Gel electrophoresis and immunoblotting

Whole cell lysates were prepared in lysis buffer (10mM Tris; pH 7.5, 4.5 mM EGTA, 0.2 mM PMSF, 1.0 mM sodium orthovanadate, 4.8 Trypsin inhibitor units of aprotinin per ml and 0.33 mM leupeptin). The supernatants were assayed for protein estimation by Folin Lowry method. The protein samples in equal amounts for a single set of experiment were analyzed by SDS- PAGE together with protein molecular weight markers (Bio-Rad) and transferred to nitro-cellulose membrane [60]. The blots were washed by Tris Buffered saline (TBS) pH 7.5 and blocked with 3% BSA (MERCK) in TBS. The blots were then probed with the primary antibody (1:500) for 4 hrs. The blots were then washed three times with TBS containing polysorbate Tween-20 (TBST) and TBS sequentially, and incubated with secondary antibody (1:1000) covalently linked to Alkaline Phosphatase for 1h. Nitro blue Tetrazolium- Bromo Chloro Indolyl Phosphate (NBT-BCIP) was used as substrate for color development (NBT-7.5 mg dissolved in 175 μl Dimethyl formamide and 75μl double distilled water. BCIP- 3.75 mg dissolved in 250μl Dimethyl Formamide).

### Densitometry analysis

Immunoblots were analyzed using a model GS-700 Imaging Densitometer and Molecular Analyst (version 1.5; Bio-Rad Laboratories).

### Measurement of cytokines by SANDWICH ELISA

The level of mouse IL-12, IFN-γ, TNF-α, IL-10 and TGF-β in the conditioned medium of melanoma cell was measured using the sandwich Enzyme-Linked Immuno-Sorbent Assay (ELISA) kit (Quantikine M; R and D systems, Minneapolis, MN, USA). The assay was performed as per the detailed instructions of the manufacturer.

### Isolation of RNA and RT-PCR

RNA was isolated according to the standard protocol [61]. Briefly total RNA extracted using TRIZOL^TM^ reagent (SIGMA). Isolated total RNA was then reverse transcribed using Revert Aid^TM^ M-MuLV Reverse Transcriptase (Fermentas). PCR amplification of the cDNA was conducted in a reaction volume of 20 μl using a Perkin Elmer Gen Amp PCR system 2400 and 0.5 unit of Taq polymerase set for 35 cycles. PCR amplified product was subsequently size fractioned on 1.5% agarose gel, stained with Ethidium Bromide and visualized under UV-light, the mRNA expression were compared, and normalized to GAPDH. The primer sequences are listed in Table 1.

**Table 1:** The primer sequences

Gene Name	Primer Sequence
Mouse IFN-γ	Forward 5′-GGATATCTGGAGGAACTGGC-3′ Reverse 5′-CGACT CCTTTTCCGCTTCCT-3′
Mouse IL-10	Forward 5′-CGGGAAGACAATAACTG-3′ Reverse 5′-CATTTCCGATAAGGCTTGG-3′
Mouse TGF-β	Forward 5′-GGATACCAACTATTGCTTCAGCTCC-3′ Reverse 5′-AGGCTCCAAATATAGG GGCAGGGTC-3′
Mouse IL-12p40	Forward 5′-CAACATCAAGAG CAGTAGCAG-3′ Reverse 5′-TACTCCCAGCTGACCTCCAC-3′
Mouse TNF-α	Forward 5′-GGCAGGTCTACTTT GGAGTCATTGC-3′ Reverse 5′-ACATTCGAGGCTCCAGTGAATTCGG-3′
Mouse GAPDH	Forward 5′-CAAGGCTG TGGGCAAGGTCA-3′ Reverse 5′-AGGTGGAAGAGTGGGAGTTGCTG-3′
Mouse Ceramide Synthase 1	Forward 5′-TGCCATCGTTTTTGCGACCA-3′ Reverse 5′-ATGTGGCGCACAATGTTTCC-3′
Mouse Ceramide Synthase 2	Forward 5′-AAGCAGGTGGAGGTAGACCTTT-3′ Reverse 5′-CATGCCAGCAACAAAGGCAAT-3′
Mouse Ceramide Synthase 3	Forward 5′-GGGTCAGTTCGTCAGTTGTTGT-3′ Reverse 5′-TGCTCTCTTGCCACTGCAAA-3′
Mouse Ceramide Synthase 4	Forward 5′-AAAGCAGGGCCCAGTTTCAA-3′ Reverse 5′-TCTTGCCCCAGCATTTTTCCTT-3′
Mouse Ceramide Synthase 5	Forward 5′-GCAATGGTGCCAACTGCAT-3′ Reverse 5′-TCCCCTGCTCTTCAGCCA-3′
Mouse Ceramide Synthase 6	Forward 5′-TTTGGCTTCCGCACAATGTCA-3′ Reverse 5′-AAGATGAGCCGCACCATGAA-3′
Mouse ASMase	Forward 5′-CTCCGCCTCATCTCTCTCA-3′ Reverse 5′- GAGTGTGGCCAAAGAACTG-3′
Mouse PKCδ	Forward 5′-ACAAATGCAGGCAATGCAACG-3′ Reverse 5′-GGCATTTGTGGTGCACATTCA-3′
Mouse IRF-1	Forward 5′- CAGAGGAAAGAGAGAAAGTCC-3′ Reverse 5′- CACACGGTGACAGTGCTGG-3′
Mouse IRF-2	Forward 5′- CAGTTGAGTCATCTTTGGGGC-3′ Reverse 5′- TGGTCATCACTCTCAGTGG-3′
Mouse IRF-3	Forward 5′- TACGTGAGGCATGTGCTGA-3′ Reverse 5′- AGTGGGTGGCTGTTGGAAAT-3′
Mouse IRF-4	Forward 5′- GTGACTGTGCCCTGGCTTAT-3′ Reverse 5′- TGGACATGATCTGGGCAACC-3′
Mouse IRF-5	Forward 5′- AATACCCCACCACCTTTTGA-3′ Reverse 5′- TTGAGATCCGGGTTTGAGAT-3′
Mouse IRF-6	Forward 5′- GATGTACGATGGCACCAAGG-3′ Reverse 5′- ACCGTTGATGTTCAGGAAGG-3′
Mouse IRF-7	Forward 5′- TGCAGAAGGTGGTGGGACA-3′ Reverse 5′- TGCTATCCAGGGAAGACACA-3′
Mouse IRF-8	Forward 5′-AACTGTGCTCTGGGCTCATC-3′ Reverse 5′- CCTCCGGGAAGTGTCCCTTA-3′
Mouse Sphk1	Forward 5′-TTTGGAGGTTGCTGACGAGGTA-3′ Reverse 5′-GCTCCTGCGTTCAGCTTCTTAT-3′
Mouse Sphk2	Forward 5′-TTCTCGATGGTATGTGGGAGGA-3′ Reverse 5′-AGCAAGCTCCGATCATGTCTCT-3′
Mouse cPLA_2_	Forward 5′-TGTGTACAATCTTTGTGTTGTTTCA-3′ Reverse 5′-CGACTCATACAGTGCCTTCATCAC-3′
Mouse TNFR1	Forward 5′-GGGCACCTTTACGGCTTCC3-3′ Reverse 5′-GGTTCTCCTTACAGCCACACA-3′
Human PKCδ	Forward 5′-AAAGGCAGCTTCGGGAAGGT-3′ Reverse 5′-TGGATGTGGTACATCAGGTC-3′
Human GAPDH	Forward 5′-GTTCGACAGTCAGCCGCATC-3′ Reverse 5′-GTTCTCAGCCTTGACGGTGC-3′

### Preparation of small interfering RNA

PKCδ (Sense 5′-AAC CTC ACT ACG CAT AGA CTG CCT GTC TC-3′), (Antisense 5′-AAC CTC ACT ACG CAT AGA CTG CCT GTC TC-3′), Sphk1 (Sense 5′-AAG AGC TGC AGA GCC TTG CCC CCT GTC TC-3′), (Antisense 5′-AAG GGC AAG GCT CTG CAG CTC CCT GTC TC-3′), Sphk2 (Sense 5′- AAG TCG CTG TAT GTG TAG GGC CCT GTC TC-3′), (Antisense 5′ AAG CCC TAC ACA TAC AGC GAC CCT GTC TC -3′) specific small interfering RNA (siRNA) were prepared using the Silencer siRNA Construction kit (Ambion) according to manufacturer's protocol. A nonspecific scrambled siRNA (control siRNA) was generated with same GC content for control.

### cPLA2 activity assay

cPLA2 activity assay was conducted using a cytosolic phospholipase A2 assay kit (Abcam, Cambridge, United Kingdom) according to the manufacturer's instructions.

### Caspase activity assay

Caspase activity assay was conducted using caspase assay kits for caspase-3, 7, 8, 9 (Abcam, Cambridge, United Kingdom) according to the manufacturer's protocol.

### Sphingosine kinase activity assay

Sphingosine kinase activity was performed according to the standard protocol as described elsewhere [62]. Briefly, Sphingosine kinase activity was measured from cell lysates in the presence of 20 μmol/L sphingosine, 1 mmol/L ATP, 10 mmol/L MgCl2, and 10 μCi/mL [3-^3^H] D-erythro-sphingosine (PerkinElmer, San Diego, USA). Sphk1 activity was determined in the presence of 0.25% Triton X-100, which inhibits Sphk2 activity. Sphk2 activity was determined with sphingosine added as a complex with 4 mg/mL BSA and 1 mol/L KCl, conditions in which Sphk2 activity is optimal and activity of Sphk1 is strongly inhibited. The reaction was performed at 37°C for 30 min and stopped on ice.

### Hypoxic treatment

To mimic hypoxic condition, melanoma cells were cultured in a humidified incubator at 37°C in an atmosphere containing 5% O_2_, 85% N_2_ and 10% CO_2_ with the help of a N_2_ and a CO_2_ gas mixture in the Stem cell technology hypoxia chamber (Canada).

### Chromatin immuno-precipitation (ChIP) assay

CHIP assays were conducted using the CHIP Assay kit following the manufacturers Protocol (Millipore, Billerica, MA) as described elsewhere [63]. With the extracted DNA, PCR was conducted using TNFα promoter specific primers, specifically IRF1 binding sites. PCR amplified product was resolved on 2% agarose gel, stained with ethidium bromide and visualized under UV-light. The primers from TNFα promoter used for PCR were: TNF - 533, 5′- CCT CCA AGA ACT CAA ACA GGG GGC TTT CCC-3’; TNF + 45, 5’-CTC CTG GCT AGT CCC TTG CTG TCC TCG CTG-3’.

### Flow cytometry analysis

Melanoma cells in different experimental conditions were harvested and washed twice in ice-cold fluorescence-activated cell sorter (FACS) buffer (PBS containing 10% (w/v) BSA and 0.1% (w/v) sodium azide). Next, cells were collected by centrifugation (2000 rpm for 5 min), and exposed to FITC-conjugated anti-mouse ceramide antibody, anti-mouse TNFα receptor antibody for 1–2 h at 4°C in the dark. For intracellular cytokine staining, brefeldin A (10 mg/ml) treated melanoma cells were permeabilized (0.1% saponin) and stained with anti-mouse TNFα-PE antibodiy. After incubation, cells were washed in FACS buffer, and the percentage of cells expressing the targets were determined by comparison of fluorescence emission intensities collected using FACS Verse (Beckton Dickinson, NJ, USA).

### Detection of cell proliferation

Melanoma cells in different condition were harvested and washed twice with PBS. Intracellular DNA was labeled with 1 ml of cold propidium iodide solution containing 0.1% Triton X- 100, 0.1 mM EDTA, 0.05 mg/ml RNase A, 50 ug/ml propidium iodide in PBS, and further incubated on ice for 30 min in the dark. Cytometric analyses were performed using a flow cytometer (FACS Verse, Beckton Dickinson, NJ, USA) and Cell Quest software.

Alternatively, (3H)-thymidine in corporation was determined. Cells were seeded in 96-well multi-plates. Each well was then pulsed with 1m Ci(3H) –thymidine (specific activity 20Ci/mmol). Sixteen hrs later, cells were washed twice with ice-cold PBS, treated with 5% trichloroaceticacid for 30 min at 4°C and solubilized with 0.5N NaOH. (3H)-Thymidine incorporation was determined on liquid scintillation counter (Wallac1409DSA, Finland). Data points for all assays were obtained in triplicate, and expressed as cpm/ug protein. Background radioactivity from cell-free wells was determined and subtracted from all data points

### *In vitro* tumor apoptosis assay

*In vitro* apoptosis of tumor cells was determined using the apoptosis detection kit (BD Pharmingen) as per the manufacturer's protocol. In brief, treated melanoma cells were incubated for 37°C in a humidified atmosphere with 5% CO_2_. After 24 hrs cells were washed with cold PBS and suspended in binding buffer and incubated with Annexin V-FITC and propidium iodide for 15 min at room temperature in the dark. Apoptotic cells were monitored by flow cytometric analysis.

### Confocal microscopy

For IRF1 translocation study, cells on cover slips were fixed with 3% paraformaldehyde for 15 min in ice. Then cells were incubated in 0.5% Triton-X in PBS (25 min, room temperature) then washed twice with PBS. Slides were blocked with 1% BSA for 1 h at room temperature, and the rabbit polyclonal IRF1 antibody (final concentration 1:200) was added to the solution and incubated for 4 h. Slides were rinsed twice and washed three times with PBS for 5 min. The secondary antibody goat anti-rabbit IgG–PE conjugate (1:500) was incubated with the cells for 1 h at room temperature, followed by washes with PBS. The nuclei were stained with DAPI present in Vectashield, which was used for mounting. Finally, the slides were visualized under a Leica TCS SP8 Confocal platform.

### Production of lentiviral particles for PKCδ and control shRNA

PKCδ shRNA (GenBank accession no. NM_011103) in pGIPZ lentivirus vector and control shRNA in pGIPZ lentivirus vector were purchased from Dharmacon (Lafayette, CO, USA). These shRNA were used for lentiviral production using Trans-Lentiviral packaging system (Dharmacon, USA) in HEK293T cell line following the manufacturer's protocol.

### Animal

Female 6-8 weeks old C57BL/6 mice were obtained from National Center for Laboratory Animal Sciences, Hyderabad, India. Mice were maintained under strict regulation with proper hygiene and every experiment was conducted in compliance with the regulations of Institutional Animal Ethics Committee.

### Tumor induction and treatment

Mice were injected (i.v) with 5 × 10^6^ transduction units (TU) of lentivirus-expressing PKCδ shRNA and control shRNA before day 3 of tumor induction. The mice were injected subcutaneously with B16F10 cells (3 × 10^5^ cells) in 100μl PBS into the right flank of each mouse. Mice were divided into 6 groups (n = 4). The control group received vehicle (PBS), while treatment group received intradermal (i.d.) injection of cisplatin (10 mg/kg bw) for 7 days after day 3 of tumor cell implantation. Tumor size was measured bi-dimensionally with calipers every 2 days, and tumor volume was estimated using the formula: (a × b^2^)/2, where a is the largest diameter and b is its perpendicular. Mice were euthanized after day 21 and excised surgically.

### Histological studies

Kidney tissues of normal mice and treated mice were fixed by immersion in 10% neutral buffered formalin for 24 h at room temperature and then embedded in paraffin. 5μm thin tissue sections were then stained with hematoxylin-eosin (HE) and pathophysiological changes were observed under bright field microscope (Leica 269Microsystem DN1000; camera: DFC450 C).

### Statistical analysis

The data, represented as mean ± standard deviation (SD), is from one experiment, which was performed at least three times. Student's *t* test was employed to assess the significance of the differences between the mean values of control and experimental groups. A *P* value of less than 0.05 was considered significant and less than 0.001 was considered highly significant.

## Abbreviations

PKCδ: protein kinase Cδ
IRF: interferon regulatory factor
TNFα: tumor necrosis factor alpha
ASMase: acid sphingomyelinase
cPLA_2_: cytosolic phospholipase A2
Sphk: sphingosine kinase
FB1: fumonisin B1
IL: interleukin
AKT: arachidonyl trifluoromethyl ketone
IFN-γ: interferon gamma
TGF-β: transforming growth factor beta
S1P: sphingosine-1-phosphate
